# *rac*-8a’-Methyl-3′,4′,8′,8a’-tetra­hydro-2′*H*-spiro­[[1,3]dioxolane-2,1′-naphthalen]-6′(7′*H*)-one

**DOI:** 10.1107/S1600536811034349

**Published:** 2011-08-27

**Authors:** Franz Werner, Liina Toon, Riina Aav

**Affiliations:** a Department of Chemistry, Tallinn University of Technology, Akadeemia tee 15, 12618 Tallinn, Estonia

## Abstract

The title racemic compound, C_13_H_18_O_3_, a common precursor in the total synthesis of terpenes, crystallizes with two molecules in the asymmetric unit. The crystal structure is made up of triple chains, formed by weak inter­molecular C—H⋯O contacts, propagating in the *a*-axis direction.

## Related literature

For the synthesis of the title compound, see: Smith *et al.* (2007 ▶). For the crystal structure of the educt, 9-methyl-Δ^5,10^-deca­lin-1,6-dione, see: Jones *et al.* (1973 ▶). For application of the title compound as a precursor in the synthesis of terpenes, see: Foot *et al.* (2006 ▶); Hatzellis *et al.* (2004 ▶); Coltart & Danishefsky (2003 ▶).
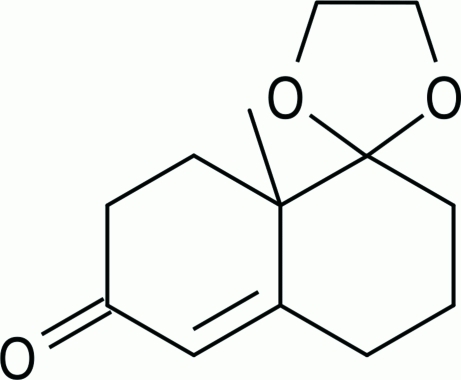


## Experimental

### 

#### Crystal data

C_13_H_18_O_3_
*M* *_r_* = 222.27
Triclinic, 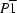

*a* = 9.6841 (15) Å
*b* = 10.5515 (14) Å
*c* = 12.8717 (19) Å
α = 102.493 (4)°
β = 111.938 (4)°
γ = 98.665 (4)°
*V* = 1151.6 (3) Å^3^
*Z* = 4
Mo *K*α radiation
μ = 0.09 mm^−1^
*T* = 200 K
0.60 × 0.40 × 0.40 mm

#### Data collection

Bruker SMART X2S diffractometer
Absorption correction: multi-scan (*SADABS*; Sheldrick, 1996 ▶) *T*_min_ = 0.948, *T*_max_ = 0.965
11107 measured reflections
4037 independent reflections
3265 reflections with *I* > 2σ(*I*)
*R*_int_ = 0.032

#### Refinement

*R*[*F* ^2^ > 2σ(*F* ^2^)] = 0.039
*wR*(*F* ^2^) = 0.106
*S* = 1.05
4037 reflections
291 parameters
H-atom parameters constrained
Δρ_max_ = 0.20 e Å^−3^
Δρ_min_ = −0.19 e Å^−3^

### 

Data collection: *GIS* (Bruker, 2010 ▶); cell refinement: *SAINT* (Bruker, 2009 ▶); data reduction: *SAINT*; program(s) used to solve structure: *SHELXS97* (Sheldrick, 2008 ▶); program(s) used to refine structure: *SHELXL97* (Sheldrick, 2008 ▶); molecular graphics: *Mercury* (Macrae *et al.*, 2008 ▶), *OLEX2* (Dolomanov *et al.*, 2009 ▶) and *VESTA* (Momma & Izumi, 2008 ▶); software used to prepare material for publication: *SHELXL97*.

## Supplementary Material

Crystal structure: contains datablock(s) global, I. DOI: 10.1107/S1600536811034349/su2308sup1.cif

Structure factors: contains datablock(s) I. DOI: 10.1107/S1600536811034349/su2308Isup2.hkl

Supplementary material file. DOI: 10.1107/S1600536811034349/su2308Isup3.cml

Additional supplementary materials:  crystallographic information; 3D view; checkCIF report

## Figures and Tables

**Table 1:** Hydrogen-bond geometry (Å, °)

*D*—H⋯*A*	*D*—H	H⋯*A*	*D*⋯*A*	*D*—H⋯*A*
C213—H2*KC*⋯O21^i^	0.99	2.52	3.450 (2)	156
C16—H16⋯O11^ii^	0.95	2.62	3.547 (2)	166
C17—H17*A*⋯O21^iii^	0.99	2.65	3.441 (2)	137
C113—H1*KC*⋯O11^iv^	0.99	2.70	3.515 (3)	140

Symmetry codes: (i) 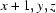; (ii) 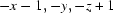; (iii) 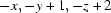; (iv) 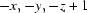.

**Fig. 1.**
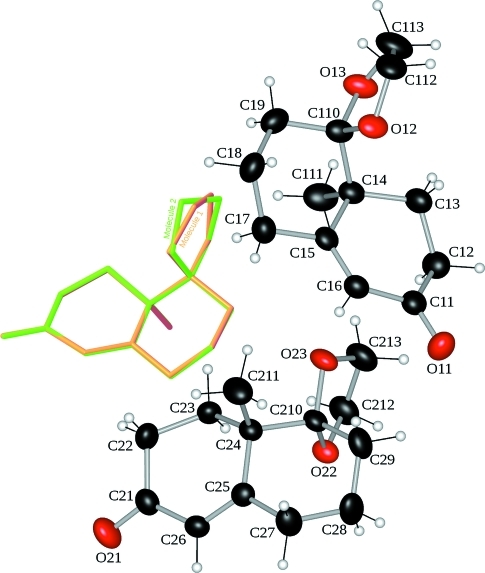
A view of the molecular structure of the two independent molecules (1 and 2) of the title compound, with the numbering scheme. Displacement ellipsoids are drawn at the 50% probability level. Inset: Overlay of the two symmetry-independent molecules (H atoms have been omitted here; Molecule 1 (orange): C11, C12, ···; Molecule 2 (green): C21, C22, ···).

**Fig. 2.**
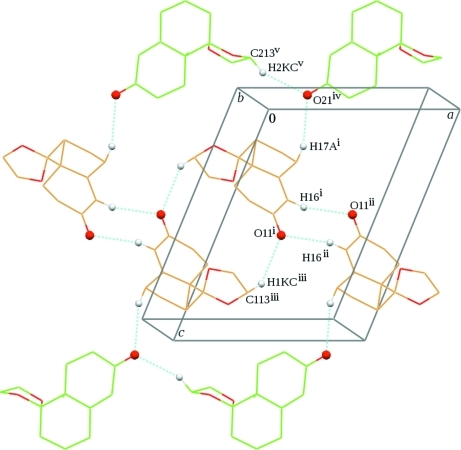
Weak C—H···O contacts in the crystal structure of the title compound [see Table 1 for details; Molecule 1 (orange), Molecule 2 (green): H atoms, except for contact atoms, have been omitted for clarity; Symmetry codes: (i) -x, -y, 1 - z; (ii) 1 + x, y, z; (iii) x, y, z; (iv) x, -1 + y, -1 + z; (v) -1 + x, -1 + y, -1 + z].

**Fig. 3.**
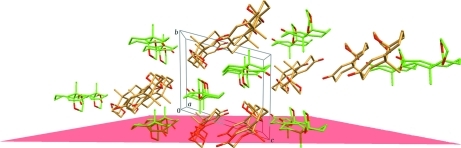
Perspective view of the crystal packing of the title compound along the a axis [Molecule 1 (orange), Molecule 2 (green)]. The orientation of the triple-chains, formed by weak C—H···O contacts, with respect to the unit cell is indicated by a red plane. H atoms have been omitted for clarity.

## supplementary crystallographic information

### Comment

The title compound crystallized at 200 K with two formula units (1 and 2), of the same handedness, in the asymmetric unit (Fig. 1). The conformation of the two molecules is nearly identical, apart from the dioxolane rings which are of opposite twist (see Inset in Fig. 1). In molecule 1 the dioxalane ring has an envelope conformation on atom O12, while in molecule 2 the envelope conformation is on atom C213. In both molecules the cyclohexane rings adopt chair conformations, while the cyclohexanone rings are somewhat flattened due to the presence of the carbonylic carbon and the double bond.

In the crystal molecules are linked by four different sets of rather weak C—H···O contacts (Fig. 2, Table 1). This results in the formation of triple-chains running along the *a* axis, with central strands of molecules of conformation 1 flanked by molecules of conformation 2 (Figs. 2 and 3).

### Experimental

The title compound was prepared from racemic Wieland–Miescher ketone according to a described procedure (Smith *et al.*, 2007), with a minor modification to the purification method. After extraction the raw product was purified by flash chromatography (2% i-PrOH in petroleum ether) and the solvent of the so obtained fractions was distilled off. The portion containing a mixture of the title compound and ethylene glycol was kept at room temperature for several weeks, whereupon colourless acicular crystals developed.

### Refinement

Except for the H atoms of the methyl groups, whose positions were determined from a difference Fourier map, H atoms were included in calculated positions and treated as riding: C—H = 0.98 (CH_3_), 0.99 (CH_2_), and 0.95 Å (CH) with *U*_iso_(H) = *kU*_eq_(C), where k = 1.5 for CH_3_ H atoms and k = 1.2 for all other H atoms.

### Crystal data

**Table:**

C_13_H_18_O_3_	*Z* = 4
*M**_r_* = 222.27	*F*(000) = 480
Triclinic, *P*1	*D*_x_ = 1.282 Mg m^−^^3^
Hall symbol: -P 1	Mo *K*α radiation, λ = 0.71073 Å
*a* = 9.6841 (15) Å	Cell parameters from 4738 reflections
*b* = 10.5515 (14) Å	θ = 2.3–25.0°
*c* = 12.8717 (19) Å	µ = 0.09 mm^−^^1^
α = 102.493 (4)°	*T* = 200 K
β = 111.938 (4)°	Needle, colourless
γ = 98.665 (4)°	0.60 × 0.40 × 0.40 mm
*V* = 1151.6 (3) Å^3^	

### Data collection

**Table:**

Bruker SMART X2S diffractometer	4037 independent reflections
Radiation source: XOS X-beam microfocus source	3265 reflections with *I* > 2σ(*I*)
doubly curved silicon crystal	*R*_int_ = 0.032
ω scans	θ_max_ = 25.1°, θ_min_ = 2.7°
Absorption correction: multi-scan (*SADABS*; Sheldrick, 1996)	*h* = −11→11
*T*_min_ = 0.948, *T*_max_ = 0.965	*k* = −12→12
11107 measured reflections	*l* = −15→15

### Refinement

**Table:**

Refinement on *F*^2^	Primary atom site location: structure-invariant direct methods
Least-squares matrix: full	Secondary atom site location: difference Fourier map
*R*[*F*^2^ > 2σ(*F*^2^)] = 0.039	Hydrogen site location: inferred from neighbouring sites
*wR*(*F*^2^) = 0.106	H-atom parameters constrained
*S* = 1.05	*w* = 1/[σ^2^(*F*_o_^2^) + (0.0516*P*)^2^ + 0.2344*P*] where *P* = (*F*_o_^2^ + 2*F*_c_^2^)/3
4037 reflections	(Δ/σ)_max_ < 0.001
291 parameters	Δρ_max_ = 0.20 e Å^−^^3^
0 restraints	Δρ_min_ = −0.19 e Å^−^^3^

### Special details

**Table:**

Geometry. All e.s.d.'s (except the e.s.d. in the dihedral angle between two l.s. planes) are estimated using the full covariance matrix. The cell e.s.d.'s are taken into account individually in the estimation of e.s.d.'s in distances, angles and torsion angles; correlations between e.s.d.'s in cell parameters are only used when they are defined by crystal symmetry. An approximate (isotropic) treatment of cell e.s.d.'s is used for estimating e.s.d.'s involving l.s. planes.
Refinement. Refinement of *F*^2^ against ALL reflections. The weighted *R*-factor *wR* and goodness of fit *S* are based on *F*^2^, conventional *R*-factors *R* are based on *F*, with *F* set to zero for negative *F*^2^. The threshold expression of *F*^2^ > σ(*F*^2^) is used only for calculating *R*-factors(gt) *etc*. and is not relevant to the choice of reflections for refinement. *R*-factors based on *F*^2^ are statistically about twice as large as those based on *F*, and *R*-factors based on ALL data will be even larger.

### Fractional atomic coordinates and isotropic or equivalent isotropic displacement parameters (Å^2^)

**Table:**

	*x*	*y*	*z*	*U*_iso_*/*U*_eq_	
O11	−0.36579 (13)	−0.13960 (12)	0.44218 (10)	0.0515 (3)	
C11	−0.26520 (17)	−0.07427 (15)	0.53959 (13)	0.0353 (4)	
C12	−0.11911 (18)	−0.11449 (16)	0.59416 (15)	0.0419 (4)	
H12A	−0.0964	−0.1670	0.5316	0.050*	
H12B	−0.1325	−0.1726	0.6425	0.050*	
C13	0.01603 (17)	0.00794 (16)	0.67085 (14)	0.0391 (4)	
H13A	0.0369	0.0598	0.6204	0.047*	
H13B	0.1089	−0.0228	0.7088	0.047*	
C14	−0.01326 (16)	0.10053 (15)	0.76622 (12)	0.0322 (3)	
C15	−0.17489 (16)	0.11996 (14)	0.71628 (12)	0.0303 (3)	
C16	−0.28429 (16)	0.04332 (15)	0.61172 (13)	0.0328 (3)	
H16	−0.3796	0.0667	0.5828	0.039*	
C17	−0.21067 (18)	0.22780 (17)	0.79299 (14)	0.0413 (4)	
H17A	−0.2196	0.1967	0.8580	0.050*	
H17B	−0.3115	0.2417	0.7461	0.050*	
C18	−0.08923 (19)	0.36159 (17)	0.84445 (14)	0.0447 (4)	
H18A	−0.0967	0.4038	0.7816	0.054*	
H18B	−0.1091	0.4226	0.9044	0.054*	
C19	0.07257 (18)	0.34259 (16)	0.90015 (13)	0.0414 (4)	
H19A	0.1493	0.4296	0.9267	0.050*	
H19B	0.0846	0.3117	0.9696	0.050*	
C110	0.10215 (16)	0.24032 (16)	0.81303 (12)	0.0340 (3)	
C111	0.0068 (2)	0.03947 (18)	0.86826 (15)	0.0474 (4)	
H1MA	−0.0214	0.0946	0.9250	0.071*	
H1MB	0.1144	0.0370	0.9070	0.071*	
H1MC	−0.0598	−0.0520	0.8374	0.071*	
O12	0.09080 (12)	0.28742 (11)	0.71548 (9)	0.0387 (3)	
O13	0.25563 (12)	0.22675 (12)	0.86454 (10)	0.0466 (3)	
C112	0.24137 (19)	0.35950 (18)	0.74068 (16)	0.0481 (4)	
H1KA	0.2594	0.4560	0.7798	0.058*	
H1KB	0.2570	0.3502	0.6679	0.058*	
C113	0.3463 (2)	0.2976 (2)	0.82021 (19)	0.0609 (5)	
H1KC	0.3867	0.2354	0.7765	0.073*	
H1KD	0.4343	0.3676	0.8850	0.073*	
O21	0.11049 (14)	0.70843 (14)	0.91102 (11)	0.0569 (4)	
C21	0.19562 (17)	0.73622 (16)	0.86470 (13)	0.0361 (4)	
C22	0.35845 (18)	0.81980 (18)	0.93451 (13)	0.0422 (4)	
H22A	0.3972	0.8107	1.0147	0.051*	
H22B	0.3613	0.9155	0.9410	0.051*	
C23	0.46228 (17)	0.77619 (16)	0.87668 (13)	0.0362 (4)	
H23A	0.4676	0.6835	0.8780	0.043*	
H23B	0.5676	0.8356	0.9227	0.043*	
C24	0.40501 (16)	0.78078 (14)	0.74922 (13)	0.0303 (3)	
C25	0.23169 (16)	0.71959 (14)	0.68281 (12)	0.0293 (3)	
C26	0.14168 (16)	0.69527 (15)	0.73754 (13)	0.0319 (3)	
H26	0.0366	0.6487	0.6909	0.038*	
C27	0.16356 (19)	0.68995 (19)	0.55151 (13)	0.0424 (4)	
H27A	0.1637	0.7756	0.5318	0.051*	
H27B	0.0552	0.6370	0.5181	0.051*	
C28	0.25155 (19)	0.6126 (2)	0.49578 (14)	0.0478 (4)	
H28A	0.2115	0.6063	0.4114	0.057*	
H28B	0.2355	0.5202	0.5024	0.057*	
C29	0.42334 (19)	0.68194 (18)	0.55581 (14)	0.0432 (4)	
H29A	0.4790	0.6283	0.5206	0.052*	
H29B	0.4404	0.7715	0.5437	0.052*	
C210	0.48576 (16)	0.69737 (14)	0.68634 (13)	0.0307 (3)	
C211	0.4437 (2)	0.92774 (16)	0.74809 (17)	0.0474 (4)	
H2MA	0.3994	0.9313	0.6671	0.071*	
H2MB	0.5558	0.9633	0.7825	0.071*	
H2MC	0.4006	0.9819	0.7940	0.071*	
O22	0.46457 (11)	0.56518 (10)	0.69896 (9)	0.0342 (3)	
O23	0.64712 (11)	0.75715 (11)	0.74433 (10)	0.0404 (3)	
C212	0.61156 (18)	0.54166 (18)	0.75591 (16)	0.0450 (4)	
H2KA	0.6152	0.4513	0.7167	0.054*	
H2KB	0.6363	0.5497	0.8394	0.054*	
C213	0.72049 (19)	0.64914 (19)	0.74471 (17)	0.0504 (5)	
H2KC	0.8229	0.6757	0.8119	0.061*	
H2KD	0.7325	0.6191	0.6709	0.061*	

### Atomic displacement parameters (Å^2^)

**Table:**

	*U*^11^	*U*^22^	*U*^33^	*U*^12^	*U*^13^	*U*^23^
O11	0.0446 (7)	0.0560 (8)	0.0396 (6)	0.0102 (6)	0.0118 (5)	−0.0010 (6)
C11	0.0356 (8)	0.0356 (8)	0.0373 (8)	0.0059 (7)	0.0185 (7)	0.0120 (7)
C12	0.0409 (9)	0.0356 (9)	0.0503 (10)	0.0126 (7)	0.0197 (8)	0.0121 (7)
C13	0.0312 (8)	0.0383 (9)	0.0489 (9)	0.0115 (7)	0.0173 (7)	0.0126 (7)
C14	0.0288 (7)	0.0356 (8)	0.0339 (8)	0.0068 (6)	0.0124 (6)	0.0157 (6)
C15	0.0318 (8)	0.0333 (8)	0.0323 (7)	0.0078 (6)	0.0173 (6)	0.0159 (6)
C16	0.0278 (7)	0.0368 (8)	0.0351 (8)	0.0094 (6)	0.0129 (6)	0.0132 (7)
C17	0.0393 (9)	0.0487 (10)	0.0374 (8)	0.0138 (7)	0.0184 (7)	0.0093 (7)
C18	0.0508 (10)	0.0426 (10)	0.0354 (8)	0.0139 (8)	0.0164 (7)	0.0035 (7)
C19	0.0431 (9)	0.0406 (9)	0.0308 (8)	0.0023 (7)	0.0100 (7)	0.0072 (7)
C110	0.0289 (8)	0.0416 (9)	0.0305 (8)	0.0059 (7)	0.0087 (6)	0.0180 (7)
C111	0.0452 (10)	0.0507 (10)	0.0469 (10)	0.0079 (8)	0.0134 (8)	0.0297 (8)
O12	0.0366 (6)	0.0430 (6)	0.0369 (6)	0.0022 (5)	0.0142 (5)	0.0208 (5)
O13	0.0284 (6)	0.0571 (7)	0.0505 (7)	0.0056 (5)	0.0091 (5)	0.0259 (6)
C112	0.0457 (10)	0.0418 (10)	0.0585 (11)	−0.0007 (8)	0.0275 (8)	0.0162 (8)
C113	0.0383 (10)	0.0721 (14)	0.0739 (13)	0.0040 (9)	0.0248 (9)	0.0291 (11)
O21	0.0482 (7)	0.0828 (9)	0.0488 (7)	0.0105 (7)	0.0316 (6)	0.0214 (7)
C21	0.0356 (8)	0.0407 (9)	0.0388 (8)	0.0134 (7)	0.0199 (7)	0.0144 (7)
C22	0.0397 (9)	0.0511 (10)	0.0296 (8)	0.0078 (8)	0.0147 (7)	0.0024 (7)
C23	0.0278 (8)	0.0430 (9)	0.0304 (8)	0.0041 (7)	0.0096 (6)	0.0045 (7)
C24	0.0291 (7)	0.0288 (8)	0.0342 (8)	0.0053 (6)	0.0152 (6)	0.0098 (6)
C25	0.0300 (7)	0.0275 (7)	0.0316 (7)	0.0116 (6)	0.0116 (6)	0.0107 (6)
C26	0.0256 (7)	0.0333 (8)	0.0349 (8)	0.0068 (6)	0.0112 (6)	0.0093 (6)
C27	0.0386 (9)	0.0596 (11)	0.0319 (8)	0.0185 (8)	0.0134 (7)	0.0174 (7)
C28	0.0480 (10)	0.0699 (12)	0.0254 (8)	0.0208 (9)	0.0148 (7)	0.0113 (8)
C29	0.0492 (10)	0.0544 (10)	0.0416 (9)	0.0204 (8)	0.0292 (8)	0.0213 (8)
C210	0.0283 (7)	0.0313 (8)	0.0369 (8)	0.0054 (6)	0.0175 (6)	0.0129 (6)
C211	0.0495 (10)	0.0315 (9)	0.0670 (12)	0.0072 (7)	0.0319 (9)	0.0147 (8)
O22	0.0310 (5)	0.0301 (6)	0.0454 (6)	0.0088 (4)	0.0180 (5)	0.0144 (5)
O23	0.0283 (5)	0.0391 (6)	0.0552 (7)	0.0036 (5)	0.0222 (5)	0.0120 (5)
C212	0.0372 (9)	0.0516 (10)	0.0562 (10)	0.0202 (8)	0.0222 (8)	0.0243 (8)
C213	0.0349 (9)	0.0589 (11)	0.0648 (12)	0.0165 (8)	0.0245 (8)	0.0228 (9)

### Geometric parameters (Å, °)

**Table:**

O11—C11	1.2277 (18)	O21—C21	1.2211 (18)
C11—C16	1.461 (2)	C21—C26	1.458 (2)
C11—C12	1.497 (2)	C21—C22	1.500 (2)
C12—C13	1.526 (2)	C22—C23	1.526 (2)
C12—H12A	0.9900	C22—H22A	0.9900
C12—H12B	0.9900	C22—H22B	0.9900
C13—C14	1.538 (2)	C23—C24	1.538 (2)
C13—H13A	0.9900	C23—H23A	0.9900
C13—H13B	0.9900	C23—H23B	0.9900
C14—C15	1.519 (2)	C24—C25	1.526 (2)
C14—C111	1.546 (2)	C24—C211	1.544 (2)
C14—C110	1.550 (2)	C24—C210	1.553 (2)
C15—C16	1.339 (2)	C25—C26	1.338 (2)
C15—C17	1.509 (2)	C25—C27	1.505 (2)
C16—H16	0.9500	C26—H26	0.9500
C17—C18	1.526 (2)	C27—C28	1.523 (2)
C17—H17A	0.9900	C27—H27A	0.9900
C17—H17B	0.9900	C27—H27B	0.9900
C18—C19	1.525 (2)	C28—C29	1.526 (2)
C18—H18A	0.9900	C28—H28A	0.9900
C18—H18B	0.9900	C28—H28B	0.9900
C19—C110	1.521 (2)	C29—C210	1.519 (2)
C19—H19A	0.9900	C29—H29A	0.9900
C19—H19B	0.9900	C29—H29B	0.9900
C110—O12	1.4209 (17)	C210—O23	1.4229 (17)
C110—O13	1.4276 (18)	C210—O22	1.4320 (18)
C111—H1MA	0.9800	C211—H2MA	0.9800
C111—H1MB	0.9800	C211—H2MB	0.9800
C111—H1MC	0.9800	C211—H2MC	0.9800
O12—C112	1.4197 (19)	O22—C212	1.4250 (18)
O13—C113	1.420 (2)	O23—C213	1.431 (2)
C112—C113	1.490 (3)	C212—C213	1.498 (2)
C112—H1KA	0.9900	C212—H2KA	0.9900
C112—H1KB	0.9900	C212—H2KB	0.9900
C113—H1KC	0.9900	C213—H2KC	0.9900
C113—H1KD	0.9900	C213—H2KD	0.9900
			
O11—C11—C16	121.98 (14)	O21—C21—C26	121.53 (14)
O11—C11—C12	121.98 (14)	O21—C21—C22	122.15 (14)
C16—C11—C12	115.96 (13)	C26—C21—C22	116.25 (13)
C11—C12—C13	111.41 (13)	C21—C22—C23	110.97 (12)
C11—C12—H12A	109.3	C21—C22—H22A	109.4
C13—C12—H12A	109.3	C23—C22—H22A	109.4
C11—C12—H12B	109.3	C21—C22—H22B	109.4
C13—C12—H12B	109.3	C23—C22—H22B	109.4
H12A—C12—H12B	108.0	H22A—C22—H22B	108.0
C12—C13—C14	113.01 (12)	C22—C23—C24	112.62 (13)
C12—C13—H13A	109.0	C22—C23—H23A	109.1
C14—C13—H13A	109.0	C24—C23—H23A	109.1
C12—C13—H13B	109.0	C22—C23—H23B	109.1
C14—C13—H13B	109.0	C24—C23—H23B	109.1
H13A—C13—H13B	107.8	H23A—C23—H23B	107.8
C15—C14—C13	110.68 (12)	C25—C24—C23	110.83 (11)
C15—C14—C111	109.19 (12)	C25—C24—C211	109.70 (13)
C13—C14—C111	110.03 (13)	C23—C24—C211	109.57 (13)
C15—C14—C110	107.85 (12)	C25—C24—C210	108.00 (11)
C13—C14—C110	109.46 (12)	C23—C24—C210	109.07 (12)
C111—C14—C110	109.59 (12)	C211—C24—C210	109.64 (12)
C16—C15—C17	120.37 (13)	C26—C25—C27	120.58 (13)
C16—C15—C14	122.92 (13)	C26—C25—C24	122.30 (13)
C17—C15—C14	116.66 (13)	C27—C25—C24	117.11 (12)
C15—C16—C11	123.62 (14)	C25—C26—C21	123.76 (13)
C15—C16—H16	118.2	C25—C26—H26	118.1
C11—C16—H16	118.2	C21—C26—H26	118.1
C15—C17—C18	113.34 (13)	C25—C27—C28	112.53 (13)
C15—C17—H17A	108.9	C25—C27—H27A	109.1
C18—C17—H17A	108.9	C28—C27—H27A	109.1
C15—C17—H17B	108.9	C25—C27—H27B	109.1
C18—C17—H17B	108.9	C28—C27—H27B	109.1
H17A—C17—H17B	107.7	H27A—C27—H27B	107.8
C19—C18—C17	111.26 (14)	C27—C28—C29	110.82 (14)
C19—C18—H18A	109.4	C27—C28—H28A	109.5
C17—C18—H18A	109.4	C29—C28—H28A	109.5
C19—C18—H18B	109.4	C27—C28—H28B	109.5
C17—C18—H18B	109.4	C29—C28—H28B	109.5
H18A—C18—H18B	108.0	H28A—C28—H28B	108.1
C110—C19—C18	110.78 (12)	C210—C29—C28	110.28 (12)
C110—C19—H19A	109.5	C210—C29—H29A	109.6
C18—C19—H19A	109.5	C28—C29—H29A	109.6
C110—C19—H19B	109.5	C210—C29—H29B	109.6
C18—C19—H19B	109.5	C28—C29—H29B	109.6
H19A—C19—H19B	108.1	H29A—C29—H29B	108.1
O12—C110—O13	106.21 (11)	O23—C210—O22	106.12 (11)
O12—C110—C19	109.37 (13)	O23—C210—C29	111.74 (12)
O13—C110—C19	110.38 (12)	O22—C210—C29	107.31 (12)
O12—C110—C14	107.96 (11)	O23—C210—C24	108.53 (11)
O13—C110—C14	109.50 (12)	O22—C210—C24	109.70 (11)
C19—C110—C14	113.15 (12)	C29—C210—C24	113.19 (13)
C14—C111—H1MA	109.5	C24—C211—H2MA	109.5
C14—C111—H1MB	109.5	C24—C211—H2MB	109.5
H1MA—C111—H1MB	109.5	H2MA—C211—H2MB	109.5
C14—C111—H1MC	109.5	C24—C211—H2MC	109.5
H1MA—C111—H1MC	109.5	H2MA—C211—H2MC	109.5
H1MB—C111—H1MC	109.5	H2MB—C211—H2MC	109.5
C112—O12—C110	106.80 (11)	C212—O22—C210	109.00 (11)
C113—O13—C110	108.60 (13)	C210—O23—C213	106.50 (12)
O12—C112—C113	104.81 (13)	O22—C212—C213	103.85 (13)
O12—C112—H1KA	110.8	O22—C212—H2KA	111.0
C113—C112—H1KA	110.8	C213—C212—H2KA	111.0
O12—C112—H1KB	110.8	O22—C212—H2KB	111.0
C113—C112—H1KB	110.8	C213—C212—H2KB	111.0
H1KA—C112—H1KB	108.9	H2KA—C212—H2KB	109.0
O13—C113—C112	105.70 (14)	O23—C213—C212	103.06 (13)
O13—C113—H1KC	110.6	O23—C213—H2KC	111.2
C112—C113—H1KC	110.6	C212—C213—H2KC	111.2
O13—C113—H1KD	110.6	O23—C213—H2KD	111.2
C112—C113—H1KD	110.6	C212—C213—H2KD	111.2
H1KC—C113—H1KD	108.7	H2KC—C213—H2KD	109.1

### Hydrogen-bond geometry (Å, °)

**Table:**

*D*—H···*A*	*D*—H	H···*A*	*D*···*A*	*D*—H···*A*
C213—H2KC···O21^i^	0.99	2.52	3.450 (2)	156.
C16—H16···O11^ii^	0.95	2.62	3.547 (2)	166.
C17—H17A···O21^iii^	0.99	2.65	3.441 (2)	137.
C113—H1KC···O11^iv^	0.99	2.70	3.515 (3)	140.

Symmetry codes: (i) *x*+1, *y*, *z*; (ii) −*x*−1, −*y*, −*z*+1; (iii) −*x*, −*y*+1, −*z*+2; (iv) −*x*, −*y*, −*z*+1.
